# Experimental Study of Fouling and Cleaning of Sintered Stainless Steel Membrane in Electro-Microfiltration of Calcium Salt Particles

**DOI:** 10.3390/membranes1020119

**Published:** 2011-05-30

**Authors:** Frank G. F. Qin, John Mawson, Xin An Zeng

**Affiliations:** 1 Research Center of Distributed Energy Systems, Dongguan University of Technology, Dongguan, Guangdong 523808, China; 2 Institute of Food, Nutrition & Human Health, Massey University, Palmerston North, 4401, New Zealand; E-Mail: A.J.Mawson@massey.ac.nz; 3 South China University of Technology, Guangzhou, Guangdong 510640, China; E-Mail: xazeng@scut.edu.cn

**Keywords:** electro-microfiltration, membrane, cleaning-in-place, sintered stainless steel

## Abstract

Sintered stainless steel (SSS) microfiltration membranes, which served as electrode directly, were used for the experiment of separating Alamin, a calcium salt and protein containing particles, found in dairy processing. Fouling and cleaning of the SSS membranes under the application of an external electric field were studied. The imposed electric field was found, diverging the pH of permeate and retentate. This in turn altered the solubility of the calcium salt and impacted the performance of electro microfiltration membrane. Using electric field as an enhanced cleaning-in-place (CIP) method in back flushing SSS membrane was also studied.

## 1. Introduction

Since fine particulate substances (colloids) acquire a *surface electric charge* when in contact with a polar medium (e.g., water), an electric double layer (EDL) [1] appears in the vicinity around the particle surface. Electro-filtration makes use of an electric field to increase the rate of filtration by providing an additional electrostatic repulsive force to the particles to mitigate fouling [2,3,4,5,6,7,8]. When the field is applied to microfiltration (MF), the process is called electro-microfiltration (EMF). This has raised interest in the field of membrane separation, especially in bio-separation, in recent years [9,10,11,12].

Sintered Stainless Steel (SSS) membrane has unique advantage in strength and chemical inertness, it can withstand harsh chemical and physical cleaning as well as high pressure back-flushing [13,14]. It is also electro conductive; this makes it convenient in establishing electric field upon the membrane module. By taking the advantage of electric repulsion, electro-filtration has potential to enhance the existing filtration processes (e.g., by increasing the throughput in existing plants or by reducing production cost), to develop new fractionated products based on differences in molecular charge, or to remove foulant.

## 2. Apparatus and Materials

**Figure 1:**
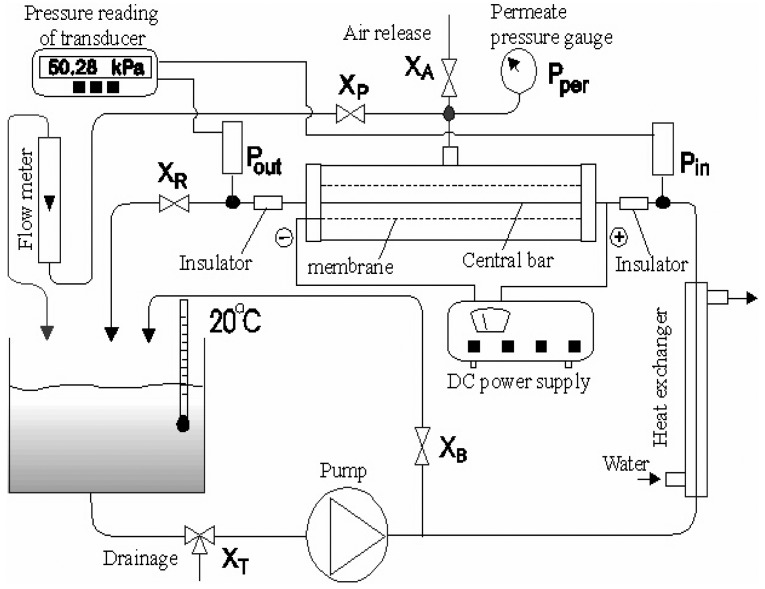
Schematic diagram of the system arrangement.

The experimental apparatus of this study comprised of a cross-flow microfiltration membrane module, a 30 L feed tank, a 0.75 kw gear pump, a tubular heat-exchanger (which maintained a constant solution temperature), 5 valves, a pair of pressure transducers (P_in_ and P_out_, 0–4 bar), which measured the trans-membrane pressure (TMP) and a XTRAVERT^®^ AC motor speed controller (mode X302, PDL Electronic LTD, Napier, New Zealand) to adjust the pump speed. A typical arrangement of the system is shown in Figure 1.

**Figure 2:**
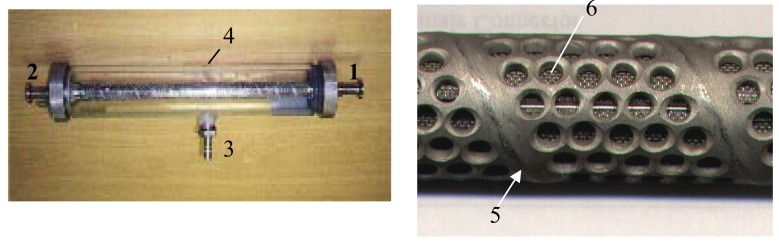
The SSS membrane module. **(1)** feed inlet; **(2)** retentate outlet; **(3)** permeate outlet; **(4)** Perspex housing; **(5)** backing of the tubular SSS membrane; **(6)** SSS membrane (under the backing).

Two SSS membranes were used in this work. They were supplied by Industrial Research Limited (NZ). The module construction, as shown in Figure 2, makes it highly resistant to organic and inorganic solvents, and can be used at high pressure (15–40 bar) and high temperature (>200 °C) [15]. The difference between the membranes was their mean pore size: 0.5 μm nominal pore size of the smaller one and 2.5 μm for the other. The tubular membrane and the central bar (also stainless steel) composed a pair of electrodes when they were electrically charged. The perspex housing collected the permeate and returned it back to the feed tank. Key dimensions of the membrane are shown in Table 1.

**Table 1:** Dimensions of two membrane modules.

Membrane tube inner diameter	ϕ13.5 mm;
Effective length	380 mm,
Central bar diameter	ϕ8 mm;
Nominal pore size	0.5 μm for the small pore size membrane, and 2.5 μm for the large pore size membrane.
Thickness	0.8 mm
Filtration area	0.019 m^2^

When the membrane and central bar were electrically charged, water could be electrolyzed with the formation of gas bubbles depending on the field strength, electrode surface properties and solution ionic strength. Thus the voltage needed to be carefully controlled. This was done by pre-setting the output current and voltage of the DC power supply (0–50 V, 0–3 A). A pair of plastic fittings was used at the inlet and outlet of the module as the electric insulator.

Two kinds of fine particles, Alamin and calcite, were used in this study. The former was a product of New Zealand Dairy Industry separated from whey [16]. It is a complex powder substance containing about 10.3% by weight protein, 25.0% by weight calcium (in phosphate) and 0.4% by weight sodium. The average particle size is 5 μm (the largest was up to 50 μm). The latter was prepared by chemical deposition. Their particle morphologies are similar and the properties are shown in Table 2. When the particles are dispersed into water in suspensions, the solution phase is rich in calcium ion in saturated state.

The major focus was on the behavior of the Alamin suspension, and the calcite was used as comparison in this work.

**Table 2:** Summary of the physical properties of calcite and Alamin powders.

	**Calcite particles**	**Alamin particles**
Appearance	white powder, particles appear to be crystalline under microscope	white powder, particles are transparent in water under the examine of microscope
Particle mean size(1) (μm)	8.15	4.63
Standard deviation(1), SD (μm)	6.46	3.53
Deposit time (2)(h)	∼1	>10
Dispersibility in water	good	good
Electrical conductivity(S/m)	--	0.003(4) 0.005(5)
Iso-electric point	--	4.7
Electrophoretic velocity(3) (μm/s)		43
Density (g/mL)	2.70–2.95	2.274(3)
Dissolving pH	5.5	4.9
Powder porosity	53%	68.8%
Chemical properties	inorganic calcium powder, dissolvable in acid solution	suspension is a amphoteric, strong buffer solution
Microbial stability	good	good in solid; perishable in suspension

Note:

(1) Number of counted particles is 200, examined with biological microscope in 10 × 40-fold.

(2) Deposit time: Time required to obtain 50% of clear water separation in a 500 mL measuring cylinder by gravity deposition.

(3) Field strength of electrophoresis: 36 V/cm, particle size: 1 μm.

(4) Electrical conductivity for the 0.07% (w/v) Alamin suspension.

(5) Electrical conductivity for the 0.7% (w/v) Alamin suspension.

Since the Zeta potential of the Alamin particles is negatively in the neutral range of pH, so they are attracted by the anode, or repulsed by the cathode, when the electric field was applied. However it was observed that only those particles smaller than 1 μm had visible electrophoretic mobility under reasonable electric field strength (e.g., 36 V/cm); larger particles did not show visible electrophoretic motion. Under this condition, in the vicinity of the anode, the electrophoretic speed of the particles around 1 μm in size were ranged from 38.17 to 49.85 μm/s in five replicates, giving an average value of 43 μm/s.

## 3. Experimental Method

The suspensions were prepared by mixing Alamin or calcite powder with deionized water, and stored in a cylindrical tank, and pumped to feed the tubular cross-flow membrane module as shown in Figure 1. The concentration of particles (Alamin or calcite) was 0.07–1% (w/v). The retentate recycled back to the tank. A by-pass stream was maintained with valve X_B_ to achieve dual functions: to agitate the suspension in the tank and to adjust the feed pressure (coupling with the adjustment of pump speed and the opening of the valve, X_R_). The pressure of the feed stream (P_in_) and retentate stream (P_out_) were measured with an inlet and an outlet transducer. The permeate pressure (P_per_) was measured with a pressure gauge. A rotermeter was used to measure the permeate flow before it returned to the tank.

The flow pattern in the membrane module was fully developed turbulence at the velocity of 2.28 m/s, which gave the Ryenolds number over 4000.

The membrane can be positively or negatively charged. However, since the fine particles in most of the actual colloidal system generally appear as negative zeta potential in a neutral pH range, a negatively charged membrane is repulsive to the deposit particles. Moreover, the cathode current on the membrane provide an electrochemical protection to the membrane from being electrochemically corroded as well. A heat exchanger was employed to maintain a constant temperature of 20 °C, and a gas-release channel (via valve V_A_) was used to minimize the gas-bubble disturbance to the rotermeter readings.

## 4. Results and Discussions

### 4.1. Membrane Polarity and Properties

**Figure 3:**
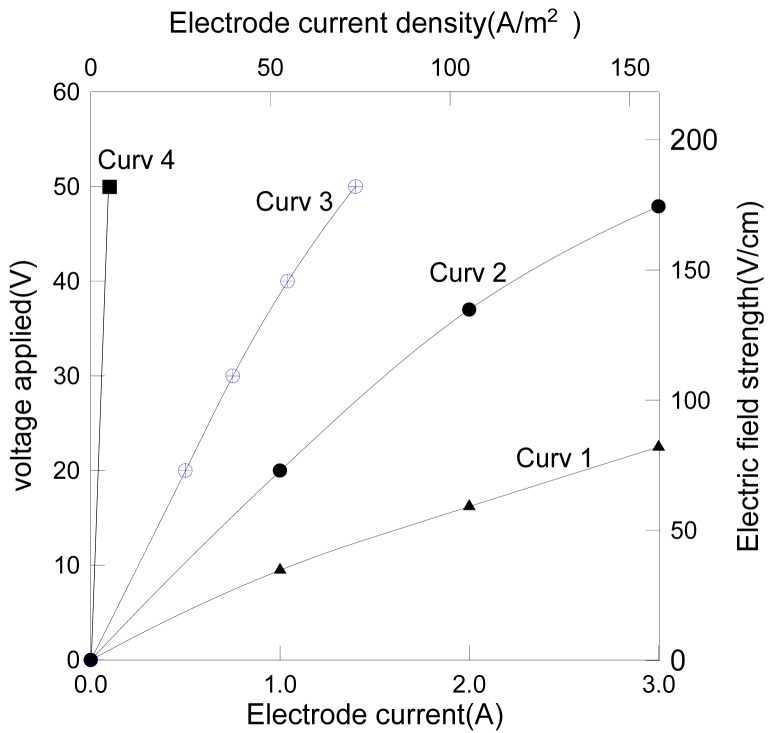
Dependence of the electrode current on the voltage applied.

The electrode current varying with the applied voltage is shown in Figure 3, where the SSS membrane worked as an anode in curve 1, but as a cathode in curve 2, 3 and 4. The calcite concentration for curve 1 and 2 was 0.7% (wt), for curve 3 was 0.07% (wt), for curve 4 was zero.

Experiments showed that (i) the electrode current was approximately proportional to the voltage applied and the particle content in the suspension; (ii) when the membrane was negatively charged as a cathode, the membrane current was twice as large as the case when it was positively charged at the same voltage. Since the membrane surface area was twice that of the central bar of the module, it implies that the electrolytic current was proportional to the cathode area. This can be explained by the fact that the hydrogen ion, a proton (H^+^), is the fastest electrophoretic ion amongst all the ions and, it was the only ion undergoing electrode reduction on the cathode in this system. Thus a larger cathode area would allow more protons to be converted to hydrogen gas in a unit time resulting in a higher electrode current. The electrode reactions are depicted by the following equations:

(1) $$2{\text{H}}_{2}\text{O}+\text{2e}\underset{\to }{\text{cathode}}{\text{H}}_{2}↑+2{\text{OH}}^{-}\;(\text{alkalization})$$

(2) $$2{\text{H}}_{2}\text{O}-\text{4e}\underset{\to }{\text{cathode}}{\text{O}}_{2}↑+4{\text{H}}^{+}\;(\text{acidification})$$

Therefore, a cathodic membrane would produce alkali permeate and acidic retentate; in contrast an anodic membrane would produce acidic permeate and alkali retentate.

By slowly increasing the applied voltage upon the SSS membrane, the voltage and field strength at which the first gas bubble appeared at the anode or cathode could be observed by using optical bio-microscopy. The results are shown in Table 3.

**Table 3:** Condition of the first gas bubble appeared.

	**Voltage (V)**	**Electric field strength (V/cm)**
On anode	∼2.5	28
On cathode	∼1	9

Note: Alamin concentration was 0.7% (w/v), pH 6.96.

If bubble formation is a sign of electrolysis, the threshold field strength found in this research is lower than many reported EMF applications [2,7,8,17]. In other words, electrolysis would be most likely to occur (more or less) in EMF/EUF(electro-ultrafiltration) for aqueous solutions.

### 4.2. EMF with a Negatively Charged Membrane

**Figure 4:**
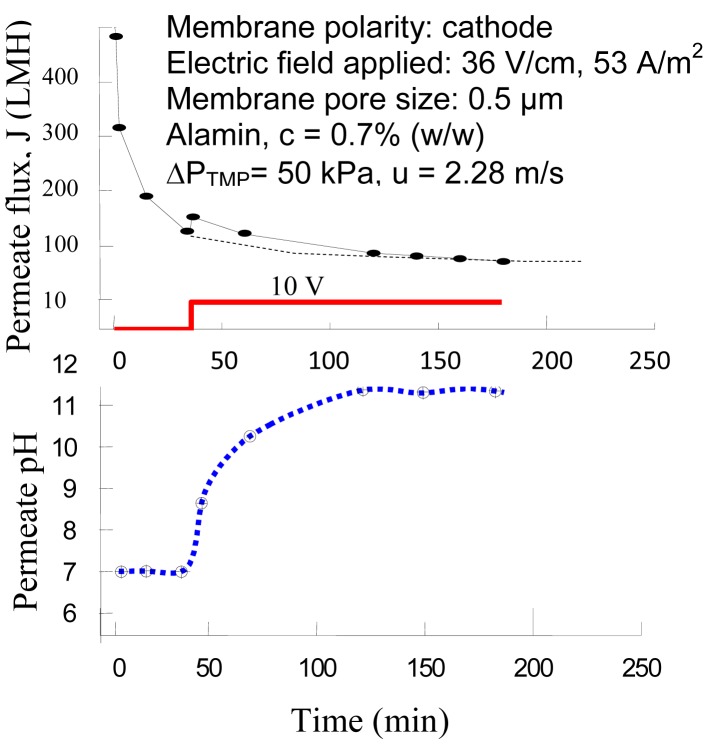
Influence of the electric field to the flux decline.

The application of EMF for separating Alamin particles with a negatively charged membrane is shown in Figure 4, in which an electric field was applied at the 40th minute after the filtration started. The observed permeate flux increased almost immediately when the voltage was applied at 10 V (DC), giving a field strength of 36 V/cm. Nevertheless, the permeate flux declined gradually after reaching the maximum. Its pH was measured as well, which increased from 7 to 11. The initial flux gain (Δ**J**) was about 25 L·m^−2^·h^−1^. The dashed line under the peak represents the possible trend of the permeate flux if the membrane was not charged. Further study revealed that the generation of hydrogen gas on the cathode membrane plays an important role in contributing the rapid increase of permeate reading, as such, the permeate alkalization was due to proton (H^+^) consumption on the cathodic membrane.

**Figure 5:**
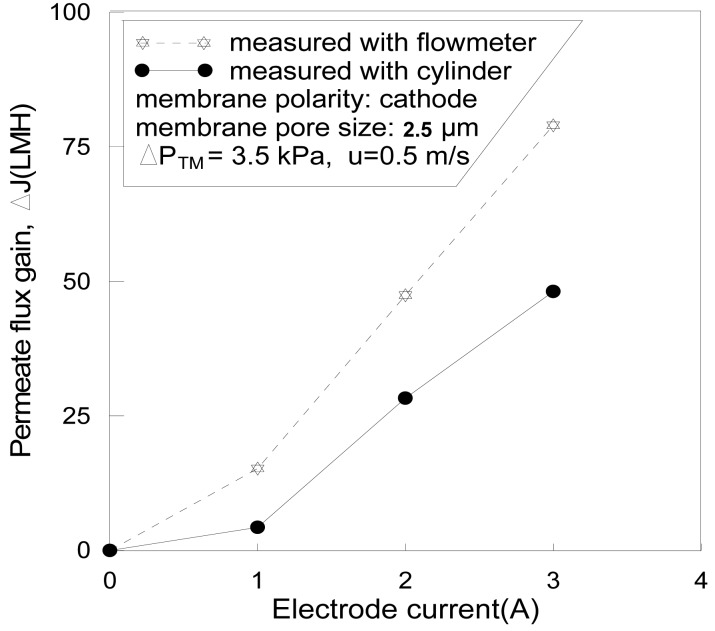
The permeate flux gain Δ**J** varies with cathode current strength.

A manually controlled pulsatile application of EMF with negatively charged membrane is shown in Figure 5. The voltage was 50 V (DC), *i.e.*, at about 180 V/cm electric field strength, and the electric charge time was 2 min. The membrane current was 1.2 A, giving a current density of 63 A/m^2^.

It was observed that the gas bubbles were formed on the cathode in permeate, passed through the rotermeter via valve Xp, as shown in Figure 1, and gave a false higher apparent permeate flux right after the power was switched on. The reason for emphasizing the bubble effect is that this seems not to be mentioned and taken into consideration by some earlier researchers [2,7,8,17].

**Figure 6:**
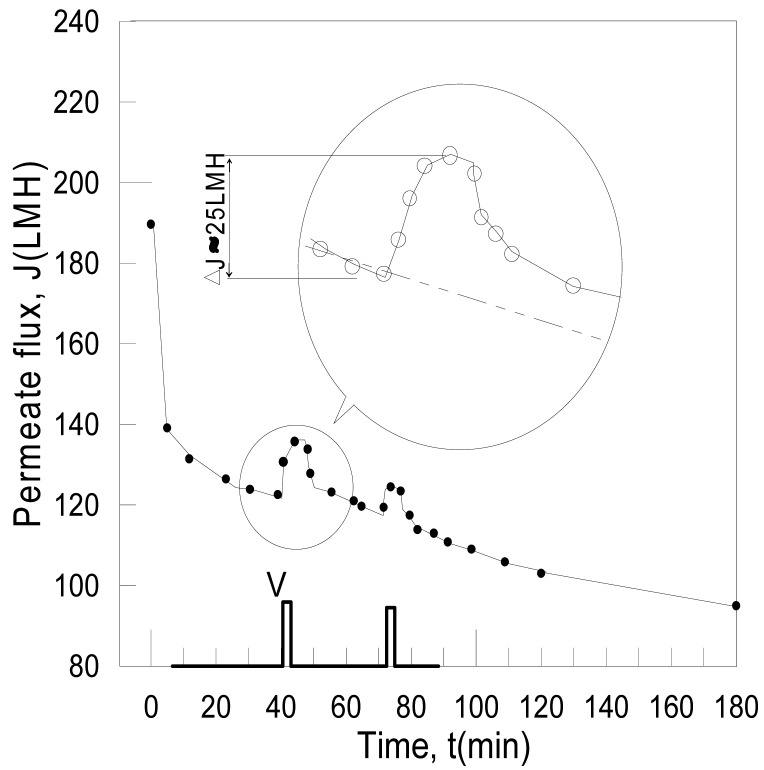
The EMF flux gain of Alamin (ΔJ) when electric field was been applied. Membrane pore size: 0.5 μm, pH = 7; ΔP_™_ = 6.5 kPa; u = 0.7 m/s; Field strength: 180 V/cm; Current I = 63 A/m^2^.

To distinguish the bubble influence from the true permeate flux, a measuring cylinder and stopwatch were used to measure the flow rate and the result was then compared with the result of the rotermeter, these are shown in Figure 6. The permeate flux gain measured with the rotermeter was about 50% higher than that measured with cylinder and stopwatch. The gas-releasing channel (valve X_A_) was closed in this run to force the gas bubbles to pass through the rotermeter (Figure 1). However, it was normally fully opened in all the other experiments of this study to eliminate the gas disturbance.

The permeate flux gain (ΔJ), shown in Figure 6, was measured at the steady state: a low transmembrane pressure (3.5 kPa) was maintained stably under which the permeate flux was lower than the *critical flux* and no obvious flux decline was observed for a long time [18,19]. After the electric field was applied, the permeate flux gradually increased and reached a maximum value in about 5–10 min. The higher the voltage, the greater the electric current, and the larger the flux gain. After the flux reached to the maximum, it gradually decreased again whether the electric field was maintained or not, thus the variation of the flux produced a peak. The flux difference between the peak and the original level is defined as the permeate flux gain, as shown in Figure 6.

**Figure 7:**
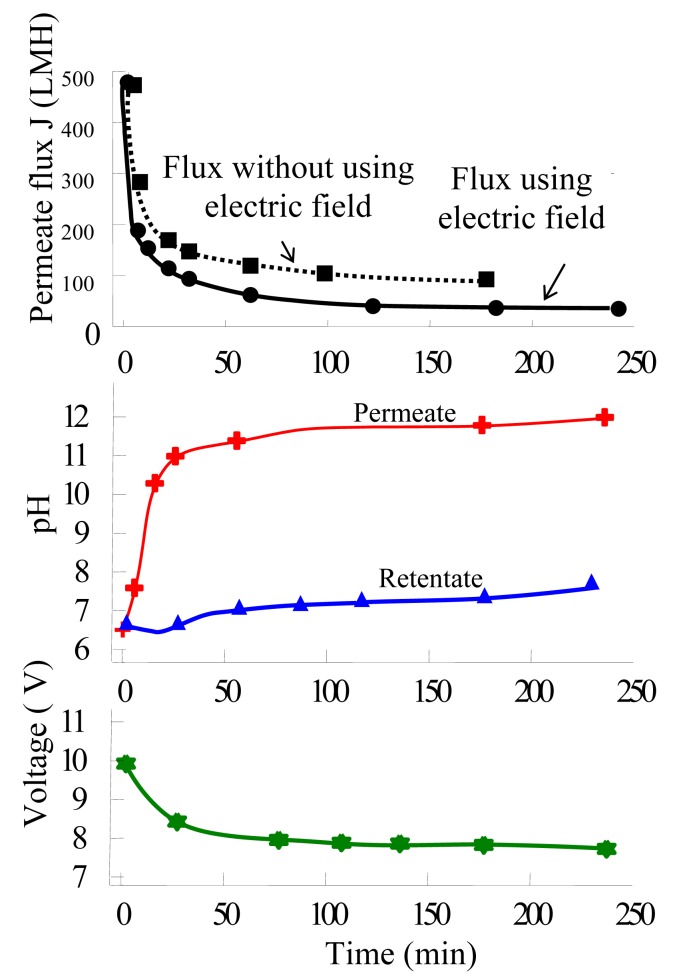
The pH divergence of the permeate and retentate in a continuous run of EMF, in which the membrane worked as a cathode (Current density: 53 A/m^2^; Alamin content c = 0.7% (w/v); ΔP_™_ = 50 kPa; u = 2.28 m/s).

As the permeate was found to be alkalizing when the SSS membrane worked as a cathode, a further experiment was carried out to trace the pH change of the permeate, retentate and bulk suspension in the storage tank, during which the permeate and retentate were both returned to the storage tank and mixed. Samples were collected every 10 min and measured with a pH meter. The result is shown in Figure 7. The permeate pH increased as usual but the retentate pH decreased in the first 20 min, and then increased as well. The whole suspension in the storage tank was pH 7.7–7.8 at the end of the experiment, slightly increased from the original pH 7.2.

As can be seen, the effectiveness of the electric field found in this research was not as good as many others in improving the permeate flux and mitigating fouling. This can be explained by the solubility of calcium salt, which decreases as the solution pH increases, especially the solution on membrane surface is subject to alkalinizing when the membrane directly served as cathode itself. Note the calcium ion was in saturated state in the liquid phase. This was different with many other reported EMF applications.

Alkalization of permeate might result in a very small amount of calcium salt came out from the solution as newly-formed tiny crystalline particles. Since the solubility of calcium salt was very low compared to the solid content of the suspension (e.g., the solubility of calcium carbonate is 1.53 × 10^−3^ g/100 g H_2_O at room temperature [20], but the solid content in the suspension for this study was ∼0.7 g/100 g H_2_O), these newborn particles would not alter the total solid content of the suspension obviously. However, since the newborn particles mainly formed close to the cathodic membrane, this must induced more fouling and resulted in decline of permeate, so as to producing a peak of the permeate flux.

The same principle may be applied to the EMF of calcite particles and other calcium salt particulate, in which the calcium solubility subjects to change in the variation of pH.

### 4.3. EMF with a Positively Charged Membrane

**Figure 8:**
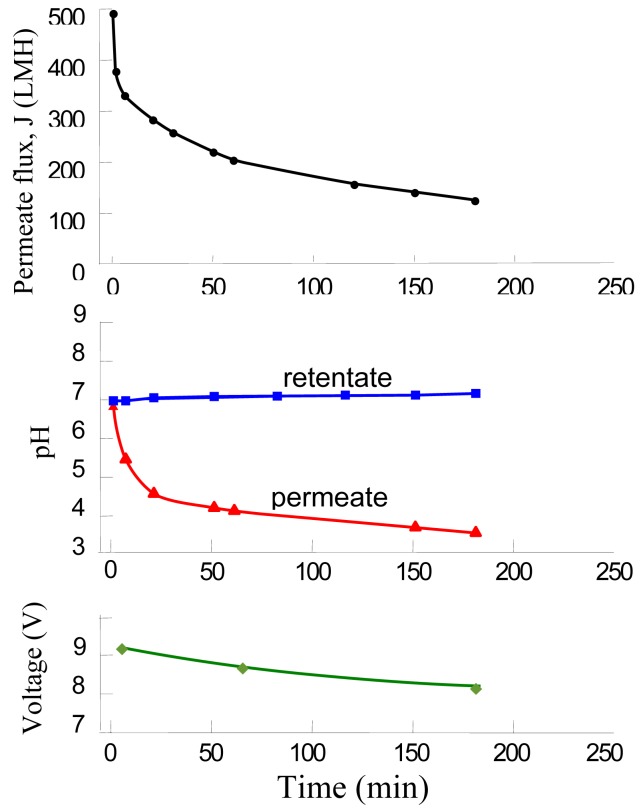
The pH divergence of the permeate and retentate in a continuous run of EMF, in which the membrane worked as an anode (Current density: 53 A/m^2^; Membrane pore size: 0.5 μm; Alamin content: c = 0.7 % (w/v); ΔP_TM_ = 50 kPa; u = 2.28 m/s).

The results of EMF experiments using a positively charged membrane are shown in Figure 8. In contrast to the negatively charged operation, the permeate pH reached a lower point (e.g., pH ≈ 3.5), and the permeate flux at 150 min was 65% higher than that of the uncharged membrane and was about 300% higher than the negatively charged membrane. The generation of hydrogen ions (H^+^) on the anode (membrane) was the reason for permeate acidification. This in turn resulted in the dissolving of calcium salt foulant and reduced membrane fouling. Despite the electrical attraction of the negatively charged particles by the membrane, the overall effect was that the permeate flux increased compared to the experiment without using an electric field.

However, when the SSS membrane was positively charged, it was subject to the risk of anodic etching and was observed under microscope in this research. Though the suspensions used in this study were all prepared with deionized water, we still found the electro conductivity of the suspension to be much greater than the deionized water itself, indicating that the Alamin and calcite particles used in this study contained little amounts of soluble salts as impurities. The electro oxidation-reduction of acid radicals on the anodic membrane could cause corrosion, so the number of trials and the time using the membrane as an anode were all limited in this study to avoid damaging the membrane.

### 4.4. Electro Cleaning in Back Flushing

It is particularly suitable to restore the filterability of the SSS membrane using back flushing plus electric field as a cleaning-in-place (CIP) method because of its mechanical strength and electrical conductivity. The gas bubbles formed underneath the foulant is effective in removing the foulant layer on the membrane surface outside the pore canal. However it is less effective for cleaning inside the pore fouling.

**Figure 9:**
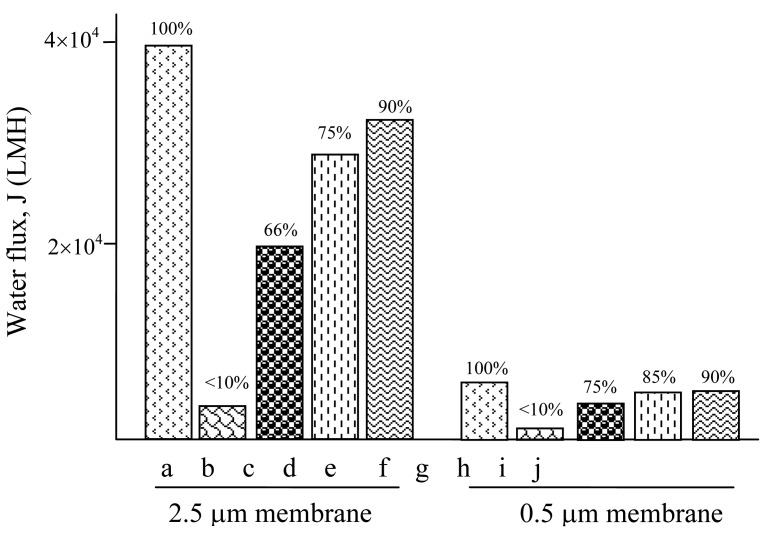
Comparison of the water flux restore after cleaning with different methods. **(a)** Original; **(b)** Fouled; **(c)** Back flush with 100 L water at 50 kPa; **(d)** Plus electric field at 50 V; **(e)** Plus 0.2% nitric acid wash for 10 min; **(f)** Original; **(g)** Fouled; **(h)** Back flush with 100 L water at 100 kPa pressure; **(i)** Plus electric field at 50 V; **(j)** Plus 0.2% Nitric acid wash for 10 min.

This explains the experimental results shown in Figure 9, where the filterability is expressed in water flux (LMH) (looks like incomplete sentence). In the first group of experiments, the water flux was restored by only 66% using back-flushing on the 2.5 μm membrane (Figure 9c). To restore more than 90% of the filterability, acid wash must be used (Figure 9e). The external electric field showed improved cleaning when it was applied during the back flush, but the water flux was not satisfactorily restored (Figure 9d).

As for the cleaning of the 0.5 μm membrane shown by the second group of experiments in Figure 9f-j, back flush restored 75% filterability, and achieved 85% if the external electric field was applied, indicating the electric field was working more effective for the small pore size membrane. On the other hand, since the particle size was 0.5 μm in this study, the above results can further be interpreted as there was more inside pore fouling in the 2.5 μm membrane than that in the 0.5 μm membrane; the electric field cleaned the external fouling better.

## 5. Conclusion

The application of EMF with SSS membrane for separating Alamin and calcite particles from the suspensions resulted in an instantaneous improvement of the permeate flux by about 20% when a 180 V/cm static electric field was applied, in which the membrane was working as a cathode to avoid being electro etched. Nevertheless, when the SSS membrane took the risk and was positively charged working as an anode, the peameate flux obtained a remarkable increase by 300% under the same field strength.

In the EMF of this work the permeate pH increased while the retentate pH decreased when the SSS membrane directly served as a cathode itself.

Though a negatively charged SSS membrane provided a repulsive Coulomb force against the concentration polarization of the particles accumulating on the membrane surface under the pressure driven microfiltration, the pH increase on the membrane surface might reduce the solubility of calcium salt, which in turn exacerbated the fouling. The process on a positively charged SSS membrane had the opposite effect, *i.e.*, the permeate flux increased. The authors considered this was associated with the dissolving of calcium salt fouling the SSS membrane. However, further verification might be required.

The capability of electrically charged SSS membrane in generating repulsive Coulomb force, bubbling to peel off the foulant scales and altering the pH on the membrane surface, was found to be very effective in CIP, which restored about 75–85% of water flux of the SSS membrane without using chemical agents.

## References

[1] Ladd M.F.C. (1986). Introduction to Physical Chemistry.

[2] Wakemam R.J. (1986). Eletriofiltration: Microfiltration plus eletrophoresis. Chem. Eng..

[3] Radovich J.M., Behnam B. (1985). Steady-state modelling of electroultrafiltration at constant concentrations. Separ. Sci. Tech..

[4] Moulik P. (1971). Physical aspects of electrofiltration. Environ. Sci. Tech..

[5] Henry J.D., Lawler L.F., AlexKuo C.H.A. (1977). A solid/liquid separation process process based on cross flow and electrofiltration. AIChE J..

[6] Akay G., Wakemam R.J. (1996). Electric field intensification of surfactant mediated separation. Chem. Eng. Res. Des..

[7] Wakemam R.J., Sabri M.N. (1995). Utilising pulsed flow electriic field in crossflow microfiltration of titania suspension. Trans. Int. Chem. Eng..

[8] Wakemam R.J., Tarleton E.S. (1986). Experiments using electricity to prevent fouling in membrane filtration. Filtrat. Separ..

[9] Bowen W.R., Filippov A.N., Sharif A.O., Starov V.M. (1999). A model of the interaction between a charged particle and a pore in a charged membrane surface. Adv. Colloid Interface Sci..

[10] Bowen W.R., Hilal N., Jain M., Lovitt R.W., Sharif A.O., Wright C.J. (1998). The effects of electrostatic interactions on the rejection of colloids by membrane pores—Visualisation and quantification. Chem. Eng. Sci..

[11] Chen X.D., Li D.X.Y., Lin S.X., Ozkan N. (2004). On-line fouling/cleaning detection by measuring electric resistance—Equipment development and application to milk fouling detection and chemical cleaning monitoring. J. Food Eng..

[12] Bowen W.R., Cao X. (1998). Electrokinetic effects in membrane pores and the determination of zeta-potential. J. Membr. Sci..

[13] Heikkinen M.S.A., Harley N.H. (2000). Experimental invetigation of sintered porous metal filters. J. Aerosol. Sci..

[14] (1995). Micro-steel Caustic Recovery System, Mem-Brine System.

[15] Neumann P. (2000). Sintered metal filters benifit from asymmetric design. Filtrat. Separ..

[16] Kawachi Y., Kawama T., Kotani M., Sato K., Tomizawa A., Dousako S. (2002). Method of making a milk calcium composition.

[17] Reeve C.J. (1997). Characterisation of an Electromicrofiltration Unit for use in Bio-Separation Processes. MS Thesis.

[18] Field R.W., Wu J.A.D., Howell B.B.G. (1995). Critical flux concept for microfiltration fouling. J. Membr. Sci..

[19] Howell J.A. (1995). Sub-critical flux operation of mecrofiltration. J. Membr. Sci..

[20] Williams M.L. (1996). Book review: CRC handbook of chemistry and physics, 76th edition. Occup. Environ. Med..

